# Association between *CYP2B6* genetic variability and cyclophosphamide therapy in pediatric patients with neuroblastoma

**DOI:** 10.1038/s41598-023-38983-0

**Published:** 2023-07-21

**Authors:** Katalin Mangó, Ferenc Fekete, Ádám Ferenc Kiss, Réka Erdős, János Tibor Fekete, Tamás Bűdi, Edit Bruckner, Miklós Garami, Tamás Micsik, Katalin Monostory

**Affiliations:** 1 Institute of Enzymology, Research Centre for Natural Sciences, Magyar Tudósok 2, 1117 Budapest, Hungary; 2 Doctoral School of Pharmaceutical Sciences, Semmelweis University, Üllői 26, 1085 Budapest, Hungary; 3 Center of Pediatrics, Semmelweis University, Tűzoltó 7-9, 1094 Budapest, Hungary; 4 Fejér County Saint George University Teaching Hospital, Seregélyesi 3, 8000 Székesfehérvár, Hungary; 5 Department of Bioinformatics, Semmelweis University, Tűzoltó 7-9, 1094 Budapest, Hungary

**Keywords:** Medical research, Genetics research, Outcomes research, Molecular medicine

## Abstract

Cyclophosphamide, an oxazaphosphorine prodrug is frequently used in treatment of neuroblastoma, which is one of the most prevalent solid organ malignancies in infants and young children. Cytochrome P450 2B6 (CYP2B6) is the major catalyst and CYP2C19 is the minor enzyme in bioactivation and inactivation pathways of cyclophosphamide. CYP-mediated metabolism may contribute to the variable pharmacokinetics of cyclophosphamide and its toxic byproducts leading to insufficient response to the therapy and development of clinically significant side effects. The aim of the study was to reveal the contribution of pharmacogenetic variability in *CYP2B6* and *CYP2C19* to the treatment efficacy and cyclophosphamide-induced side effects in pediatric neuroblastoma patients under cyclophosphamide therapy (N = 50). Cyclophosphamide-induced hematologic toxicities were pivotal in all patients, whereas only moderate hepatorenal toxicity was developed. The patients’ CYP2B6 metabolizer phenotypes were associated with the occurrence of lymphopenia, thrombocytopenia, and monocytopenia as well as of liver injury, but not with kidney or urinary bladder (hemorrhagic cystitis) toxicities. Furthermore, the patients’ age (< 1.5 years, P = 0.03) and female gender (P ≤ 0.02), but not CYP2B6 or CYP2C19 metabolizer phenotypes appeared as significant prognostic factors in treatment outcomes. Our results may contribute to a better understanding of the impact of CYP2B6 variability on cyclophosphamide-induced side effects.

## Introduction

Neuroblastoma is one of the most prevalent solid organ malignancies in infants and young children, mainly under 5 years of age (6% of all childhood cancers), and its incidence rates vary between 3 and 15 per million children^1,2^. It develops from neural crest cell precursors and forms the primary tumour in the adrenal medulla or along the sympathetic nervous chain. The risk-stratified treatment approach is based on the outstanding heterogeneity of the disease regarding the clinical characteristics or the biological and histological features of the tumours^3^. The therapy for patients diagnosed with low-to-intermediate-risk neuroblastomas ranges from observation alone to surgical resection of the tumour with or without moderate multiagent chemotherapy, and it is predicted to result in favourable outcome with 90–95% survival rates^4^. The treatment of patients with high-risk neuroblastomas is strictly defined by standard regimens, including induction chemotherapy, surgical resection, consolidation and maintenance therapy. However, the long-term survival rate of these patients is only 40–50% despite complex multimodal therapy^5,6^. Multiagent conventional chemotherapy is pivotal in the treatment of neuroblastoma, and cyclophosphamide is one of the most frequently used agents^3,6^.

Cyclophosphamide is an oxazaphosphorine prodrug, and metabolic activation is required for the formation of the cytotoxic nitrogen mustard. Phosphoramide mustard is an alkylating agent that creates covalent linkages, intra- and interstrand DNA crosslinks between intracellular nucleophiles, resulting in cell death^7,8^. Biotransformation of cyclophosphamide is catalysed by hepatic cytochrome P450 (CYP) enzymes, which are crucial for the bioactivation and formation of the active metabolite as well as in the inactivation of cyclophosphamide. Approximately 70–80% of the administered dose is metabolized to 4-hydroxycyclophosphamide primarily by CYP2B6 and to a minor extent by CYP2C19 and CYP3A4 enzymes, whereas only 10% of cyclophosphamide is inactivated via *N-*dechloroethylation by CYP3A4. The 4-hydroxy metabolite and its tautomer isoform, aldophosphamide are considered to be the transport form of the nitrogen mustard^9–11^. Chemical decomposition of aldophosphamide leads to the formation of the active metabolite, phosphoramide mustard, and the toxic byproduct, acrolein, while aldehyde dehydrogenase (ALDH1A1) converts aldophosphamide to the inactive excretory metabolite, carboxyphosphamide^12,13^. A sufficient response to cyclophosphamide treatment has been assumed in tumour cells with low ALDH activity, whereas high ALDH expression is thought to be associated with cyclophosphamide resistance^14,15^. The cytotoxicity of phosphoramide mustard, as a side effect of cyclophosphamide treatment, is primarily manifested in sensitive normal cell populations with low ALDH1A1 expression, especially in hematopoietic progenitor cells. Suppression of hematopoietic cell generation leading to leukopenia is relatively common after cyclophosphamide treatment^16,17^. Although CYP-mediated metabolic pathways also produce chemotherapeutically inactive metabolites, acrolein and chloroacetaldehyde are responsible for clinically significant side effects. Bladder toxicity causing hemorrhagic cystitis is one of the most common side effects associated with highly reactive, unsaturated aldehyde acrolein excreted in the urine. Cyclophosphamide-induced hepatotoxicity has been reported to occur rarely and mainly with high-dose therapy; however, it is also associated with the formation of acrolein^18–20^. Oxidative stress evoked by acrolein is efficiently prevented by the co-administration of mesna (2-mercaptoethane sulfonate) which interacts with acrolein to produce a non-toxic adduct^21,22^. The minor inactivation pathway of cyclophosphamide leads to the cleavage of the chloroacetaldehyde metabolite which has been reported to be responsible for neuro-, cardio- and nephrotoxicity^10,23,24^. A recent study has also demonstrated that the urotoxicity of chloroacetaldehyde contributes to urothelial dysfunction^25^.

Substantial variability in cyclophosphamide pharmacokinetics has been reported in patients, and the variation in the exposure to active and inactive metabolites may lead to differences in patients’ response to cyclophosphamide and in the development of adverse reactions^19,20,26–29^. The outcomes of cyclophosphamide therapy are well documented in adult patients^30^; however, only a few studies have focused on pediatric malignancies^20,31–35^. Cyclophosphamide clearance is more intense in children than in adults; furthermore, children display age-dependent response to cyclophosphamide requiring modification of dosing protocol from the very early to late childhood^27,34,35^. One of the most notable sources of interindividual variability in response to cyclophosphamide is drug metabolism, highlighting the outstanding role of CYP2B6 enzyme in both the bioactivation and inactivation pathways^30^. CYP2B6 function is primarily influenced by genetic polymorphisms, whereas non-genetic factors (e.g., medication, nutrition, age, disease) can transiently modify the expression and/or the activity of CYP2B6 enzyme^36–38^ (https://www.pharmvar.org/gene/CYP2B6, access date: 26.04.2023). In the last two decades, the impact of *CYP2B6* genetic variants was investigated on pharmacokinetics and therapeutic outcomes of several CYP2B6-substrate drugs, including efavirenz, bupropion, methadone, *S-*mephenytoin and cyclophosphamide^30,39–41^. The Clinical Pharmacogenetics Implementation Consortium (CPIC) guideline for efavirenz dosing has recently been published for patients with various CYP2B6 metabolizer phenotypes (poor, intermediate, normal and rapid/ultra-rapid metabolizers) predicted from their *CYP2B6* genotypes^42^. CYP2B6*6, one of the most prevalent allelic variants (carrying both g.18053A>G [rs2279343] and g.15631G>T [rs3745274]) is associated with decreased mRNA expression and enzyme activity, designating ’poor’ or ’intermediate’ metabolizer phenotypes. Lower clearance of efavirenz or cyclophosphamide has been reported in patients who carry CYP2B6*6 than in non-carriers^35,42–44^. CYP2B6*9 variant (g.15631G>T [rs3745274]) is associated with decreased bupropion and efavirenz hydroxylation assuming ‘poor’ or ‘intermediate’ metabolizer phenotypes similarly to CYP2B6*6; however, the clinical importance of CYP2B6*9 can be hardly interpreted because of the low prevalence in all populations^45,46^. CYP2B6*4 allele (carrying g.18053A>G [rs2279343]) creates a structurally altered enzyme variant which is associated with enhanced CYP2B6 catalytic activity, predicting ’rapid/ultra-rapid’ metabolizer phenotype^42,47^. CYP2B6*5 allele (g.25505C>T [rs3211371]) has been suggested to have a mild or negligible effect on CYP2B6 catalytic activity, and it is associated with ’normal’ metabolizer phenotype^40,42,48^. The g.-82T>C (rs34223104) single nucleotide variation (SNV) in CYP2B6*22 allele appears to enhance the transcription of *CYP2B6* gene leading to increased mRNA expression and catalytic activity, and carriers of CYP2B6*22 are categorized as ’rapid/ultra-rapid’ metabolizers^42,49–51^. Several *CYP2B6* alleles have been clearly demonstrated to result in decreased or increased CYP2B6 activity; however, the association between *CYP2B6* genetic polymorphisms and cyclophosphamide pharmacokinetics or clinical outcomes of cyclophosphamide therapy is often controversial^34,44,52–57^. The *CYP2B6* genotype–phenotype mismatch is partly explained by non-genetic factors, such as co-medications, sex and age, which can mask the effect of *CYP2B6* allelic variants. Multidrug therapy with CYP2B6 inducers (e.g., steroids) or inhibitors (e.g., thiotepa, amlodipine, ticlopidine) is a potential source of CYP2B6 phenoconversion that can transiently alter the biotransformation rates of CYP2B6 substrates^34,37,38,56,58^. It has been reported that cyclic dosage of cyclophosphamide appears to induce its own metabolism by increasing CYP2B6 protein expression in a concentration-dependent manner^10,59^.

Although the association of CYP pharmacogenetics with cyclophosphamide pharmacokinetics and cyclophosphamide-related toxic events has been studied in patients with several cancer types (e.g., breast cancer, chronic lymphoid leukaemia, non-Hodgkin’s lymphoma)^30,41,43,44^, it has been scarcely investigated in patients with neuroblastoma^28,29,35^. The major aim of the present study was to determine the role of *CYP2B6* pharmacogenetic variability and patient-specific phenoconverting factors, such as age and sex, in the development of toxic events in pediatric patients with neuroblastoma undergoing cyclophosphamide therapy. A further aim was to find any association between the therapeutic outcome and drug-metabolizing capacity of CYP2B6, the major and CYP2C19, the minor catalysts of cyclophosphamide metabolism. Our results may contribute to a better understanding of the impact of CYP variability on the clinical manifestations of cyclophosphamide treatment in children.

## Materials and methods

### Patients and data collection

Pediatric patients (N = 50) treated with neuroblastoma at the Center of Pediatrics, Semmelweis University (Budapest) were enrolled in the present retrospective study. The inclusion criteria were written informed consent from the patients’ legal representatives (generally from their parents), patients less than 18 years of age, and cyclophosphamide therapy through at least three cycles. The study was approved by the Hungarian Committee of Science and Ethics, Medical Research Council, and was conducted according to the regulations of Act CLIV of 1997 on Health and Decree 23/2002 of the Minister of Health of Hungary, and in accordance with the Declaration of Helsinki. All patients belonged to the Caucasian population, and their demographic and clinical data were recorded (Table 1). The patients were designated as low-risk or high-risk subjects using International Neuroblastoma Staging System (INSS). High-risk neuroblastoma defined as (#1) Stage M neuroblastoma (distant metastatic tumours except for Ms) above 365 days of age at diagnosis (no upper age limit) and Ms neuroblastoma (metastases confined to the skin, liver and/or bone marrow) 12–18 months old, any *MYCN* status or (#2) L2 (locoregional tumour with the presence of one or more image-defined risk factors), M or Ms neuroblastoma any age, with *MYCN* amplification, or focal high level MYC or MYCL amplification. The expression of the cellular oncogene MYCN is high in developing tissues that normally give rise to neuroblastoma, whereas the amplification of MYC and MYCL is uncommon in neuroblastoma^60,61^. Cyclophosphamide dosing was calculated from the patients’ body surface (bodyweight and height) according to the principles of standard regimen protocols considering pretreatment risk stratification^62^ (Table 1). Relevant clinical, *CYP2B6* and *CYP2C19* genotype data of neuroblastoma patients were summarized in Supplementary Table 1.

**Table 1:** Demographic and clinical data of patients.

Parameter	
Number of patients	50
Age at diagnosis (years)^a^	2.32 (0.10; 15.8)
< 1.5	16
> 1.5	34
Sex (female/male)	21/29
Bodyweight (kg)^a^	12.35 (0.59; 53.6)
Risk stratification of patients	
Low-risk	15
High-risk	35
Cyclophosphamide dose (mg/kg/day)	
Rapid COJEC^b^	10.5–25.5
Infant CO or CADO^b^	0.8–2
CADO^b^ (≥ 1.5 years old)	2.2–2.6
Therapeutic outcome: responders	
Complete remission (CR)	16
Partial remission (PR)	8
Therapeutic outcome: non-responders	
Stable disease (SD)	9
Progressive disease/exit (PD-Exit)	17

^a^median (min; max); ^b^COJEC cisplatin-vincristine-carboplatin-etoposide-cyclophosphamide, *CO* cyclophosphamide-vincristine, *CADO* cyclophosphamide-adriamycin-vincristine.

### Therapeutic outcome and treatment-related toxicity

The patients’ response to therapy (responders: complete remission and partial remission; non-responders: stable disease and progressive disease/exit) was defined according to the principles of the International Neuroblastoma Response Criteria (INRC)^63^. Hepatic, renal and hematologic toxicities were characterized by increased levels of serum alanine aminotransferase (ALT), gamma-glutamyltransferase (GGT), creatinine, sodium and potassium, and by decreased counts of leukocytes, platelets, neutrophil granulocytes, monocytes, eosinophil granulocytes and red blood cells as well as by blood in urine. These parameters were recorded a day before and at the peak or nadir (generally 7–15 days) after the cyclophosphamide treatments. Grades of renal, hepatic and bone marrow toxicities were evaluated according to the principles of National Cancer Institute Common Toxicity Criteria (CTC) version 2.0 document (Supplementary Table 2). A treatment-related increase or decrease in serum parameters and cell counts was considered when the patients’ parameters exceeded the upper limit or were below the lower limit in the normal reference populations at the same age.

### *CYP* genotyping

Blood samples of patients (N = 50) were used to determine *CYP2B6* single nucleotide variations (SNVs) [g.18053A>G (rs2279343), g.15631G>T (rs3745274), g.25505C>T (rs3211371) and g.-82T>C (rs34223104)] and *CYP2C19* SNVs [g.19154G>A (rs4244285), g.17948G>A (rs4986893), g.1A>G (rs28399504) and g.-806C>T (rs12248560)]. Genomic DNA templates were isolated from blood samples using Quick-DNA™ Miniprep Plus Kit (Zymo Research, Irvine, CA). *CYP2B6* SNVs (g.15631G>T, g.25505C>T and g.-82T>C) were identified using TaqMan™ Drug Metabolism Genotyping Assays (C_7817765_60, C_30634242_40, C_27830964_10, Thermo Fisher Scientific, Waltham, MA) according to the manufacturer’s instructions. For the analysis of g.18053A>G SNV, a two-step PCR assay based on the ‘nested’ PCR method with ‘touchdown’ thermal cycling protocol and TaqMan PCR was carried out^38^. PHASE software v2.1.1 analysis was performed from SNV data for reconstruction of *CYP2B6* haplotypes^64,65^. *CYP2C19* SNVs and haplotypes were determined by TaqMan allele-discrimination using primers and probes for g.19154G>A, g.17948G>A, g.1A>G and g.-806C>T as previously described by Kiss et al.^66^. According to the CPIC guideline regarding the functional impact of *CYP2B6* polymorphisms and *CYP2C19* diplotype to phenotype translation by PharmVar (Pharmacogene Variation Consortium), patients were classified as poor, intermediate, normal and rapid/ultrarapid metabolizers^42^ (https://www.pharmgkb.org/page/cyp2c19RefMaterials, access date: 26.04.2023).

### Data analysis

Demographic and clinical data of 50 neuroblastoma patients were collected to evaluate the association between *CYP2B6* and *CYP2C19* genetic polymorphisms and the outcome or adverse effects of cyclophosphamide treatment using InStat v3.06 (GraphPad Software, Inc., San Diego, CA). Binary logistic regression models were applied to evaluate the association between adverse effects or patients’ response to cyclophosphamide therapy as dependent variables and *CYP2B6* SNVs, haplotypes, sex and age (age categories: < 1.5 or > 1.5 years) as co-variates. Multivariate binary logistic regression analyses were performed using IBM SPSS Statistics software [v28.0.1.0 (142), IBM Corp., Armonk, NY]. In general, P value < 0.05 was considered to be statistically significant. No formal sample size estimation applied, although for logistic regression models, the general rule of including at least 10 observations/predictor variables was applied as sample size estimation^67^. Posterior power calculation was performed using G*Power 3.1.9.7 software (Christian Albrechts University, Kiel, Germany)^68^. Goodness of fit of logistic regression models was approved by Hosmer–Lemeshow test; furthermore, Akaike’s Information Criterion (AIC) and Bayesian Information Criterion (BIC) numbers were added to each model.

### Informed consent

Written informed consent was obtained from the patients’ legal representatives.

## Results

### *CYP2B6* and *CYP2C19* genetic variability in neuroblastoma patients

Clinically relevant *CYP2B6* [g.18053A>G (rs2279343), g.15631G>T (rs3745274), g.25505C>T (rs3211371) and g.-82T>C (rs34223104)] and *CYP2C19* SNVs [g.19154G>A (rs4244285), g.17948G>A (rs4986893), g.1A>G (rs28399504), g.-806C>T (rs12248560)] most frequent in Caucasian populations were identified in pediatric patients with neuroblastoma (N = 50) (Table 2). Patients who did not carry any of the *CYP2B6* or *CYP2C19* polymorphisms were considered to have wild-type CYP2B6*1 and CYP2C19*1 alleles. The prevalence of CYP2B6*6 and CYP2B6*5 alleles was relatively high in patients (22.0% and 11.0%, respectively), whereas CYP2B6*4 and CYP2B6*22 alleles occurred sporadically (5.0% and 1.0%, respectively), and no patient with CYP2B6*9 allele was identified. For *CYP2C19*, the most common allelic variants were CYP2C19*2 and CYP2C19*17 with a prevalence of 15.3% and 20.4%, respectively, whereas CYP2C19*3 and CYP2C19*4 did not occur in the children in the present study. The relative frequencies of the *CYP2B6* and *CYP2C19* alleles in neuroblastoma patients were similar to those previously published in Caucasian populations (Table 2)^36,46,69^ (https://www.pharmgkb.org/page/cyp2b6RefMaterials, access date: 26.04.2023; https://www.pharmgkb.org/page/cyp2c19RefMaterials).

**Table 2:** *CYP2B6* and *CYP2C19* allele and genotype frequencies, and genotype based phenotypes of neuroblastoma patients (N = 50).

	N^a^	Frequency (%)	
		Neuroblastoma patients	Caucasian population^b^
CYP genotype based phenotype estimation			
* CYP2B6* alleles			
*4	5	5.0	2.2–6.2
*5	11	11.0	9–12.2
*6	22	22.0	7–28.1
*9	0	0	0–4.4
*22	1	1.0	1.4–2.4
Poor/intermediate CYP2B6 metabolizer (N = 20)			
* CYP2B6* genotypes			
*1/*6	14	28.0	22.1–22.9
*6/*6	2	4.0	5.4–7.3
*5/*6	2	4.0	5.3–7.3
*4/*6	2	4.0	< 2.1
Normal/rapid CYP2B6 metabolizer (N = 30)			
*1/*1	17	34.0	21–24.1
*1/*5	9	18.0	7.3–11.3
*1/*4	3	6.0	1–4
*1/*22	1	2.0	1.3–2.1
* CYP2C19* alleles^c^			
*2	15	15.3	6.0–15.0
*3	0	0	< 1
*4	0	0	< 1
*17	20	20.4	21.5–25.0
Poor/intermediate CYP2C19 metabolizer (N = 12)			
* CYP2C19* genotypes^c^			
*1/*2	6	12.2	18.3–18.5
*2/*2	3	6.1	2.1
*2/*17	3	6.1	6.3
Normal CYP2C19 metabolizer (N = 23)			
*1/*1	23	46.9	39.0–40.5
Rapid/ultrarapid CYP2C19 metabolizer (N = 14)			
*1/*17	11	22.4	26.9–27.5
*17/*17	3	6.1	4.6–4.7

^a^number of alleles or number of patients. ^b^Based on PharmVar (https://www.pharmgkb.org/page/cyp2b6RefMaterials, https://www.pharmgkb.org/page/cyp2c19RefMaterials), Zanger^36^, Ionova^69^, Zhou^46^. ^c^Missing CYP2C19 data for 1 patient.

CYP2B6 functions and phenotypes were estimated on the basis of *CYP2B6* genotypes according to Desta et al. and PharmVar^42^ (https://www.pharmgkb.org/page/cyp2b6RefMaterials). More than one third of the patients (N = 20/50) were predicted to be poor/intermediate CYP2B6 metabolizers, whereas the majority of the patients (N = 30/50) were found to be normal/rapid CYP2B6 metabolizers. For the estimation of CYP2C19 phenotypes, the recommendations of PharmVar were followed (https://www.pharmgkb.org/page/cyp2c19RefMaterials). Poor/intermediate CYP2C19 metabolizers (N = 12/49) carried at least one copy of CYP2C19*2 allele. The patients with CYP2C19*1/*1 genotype were considered to be normal CYP2C19 metabolizers (N = 22/49), whereas those carrying CYP2C19*1/*17 or CYP2C19*17/*17 genotypes belonged to the rapid/ultrarapid CYP2C19 metabolizer category (N = 15/49).

### Patients’ *CYP2B6* genotype and cyclophosphamide-induced hepatorenal and bladder toxicity

The symptoms of hepatic, renal and bladder toxicity were followed in neuroblastoma patients during at least three cycles of ’low’ (1–2.6 mg/kg/day) or ’intermediate’ (10.5–38 mg/kg/day) doses of cyclophosphamide^17^. Toxicities were graded according to the Common Toxicity Criteria of the National Cancer Institute (CTC version 2.0; Supplementary Table 2). Elevated serum ALT and GGT levels indicated mild (grade 1 or grade 2) hepatic injury in more than half of the patients (27/49), whereas moderate (grade 3) toxicity was observed in 2 patients (2/49). Cyclophosphamide-induced bladder injury was evaluated on the basis of bloody urine symptoms, while renal toxicity was estimated by an increase in serum creatinine, sodium and potassium levels. A mild increase in serum creatinine concentrations (grade 1) indicating renal toxicity was observed in only two patients (2/49), whereas elevated sodium and/or potassium concentrations developed in 28.6% of the children (14/49). Bladder injury occurred sporadically (3/49) and was reported immediately in the first and second cycles of therapy, whereas in subsequent cycles, bloody urine symptoms were not observed.

CYP2B6 function has been supposed to be related to the development of cyclophosphamide-induced side effects; therefore, the association of patients’ *CYP2B6* genotype-based phenotypes with hepatic, renal and bladder toxicity was evaluated (Table 3). In 26.3% of poor/intermediate CYP2B6 metabolizers (5/19), serum ALT levels exceeded the upper limit of normal reference population, while more than half of the patients with normal/rapid CYP2B6 metabolizer phenotypes displayed elevated serum ALT levels (60.0%, 18/30). This means that CYP2B6 phenotype significantly contributed to the development of hepatic injury, as indicated by ALT increase (OR 0.238; 95% CI 0.068–0.835; N = 49, P = 0.03) (Table 3). Multivariate binary logistic regression analysis with *CYP2B6* SNVs, haplotypes or genotype-based phenotypes and non-genetic factors, including age and sex as independent variables, identified that the incidence of abnormal serum ALT concentrations was significantly lower in poor/intermediate metabolizers than in normal/rapid metabolizers (P = 0.02) most probably due to CYP2B6*6 (g.-82T/15631T/18053G/25505T) allele (P = 0.008). However, *CYP2B6* SNVs and non-genetic factors (sex, age) had no influence on ALT increase (Table 4). Furthermore, no significant association was observed between CYP2B6 function and abnormal serum GGT levels induced by the therapy (poor/intermediate CYP2B6 metabolizers 21.0% *vs* normal/rapid CYP2B6 metabolizers 40.0%, OR 0.400; 95% CI 0.106–1.502; P = 0.21) (Table 3). Blood in urine, the symptoms of bladder injury, and increased serum creatinine concentrations indicating renal toxicity rarely occurred in patients (3/49 and 2/49, respectively) (Table 3), and these patients were predicted to have normal CYP2B6 metabolizer phenotype (CYP2B6*1/*1, CYP2B6*1/*5). The incidence of abnormal serum sodium and potassium levels in poor/intermediate CYP2B6 metabolizers was equal to those in normal/rapid CYP2B6 metabolizer subjects (sodium: 5.2% *vs* 6.6%; OR 0.778; 95% CI 0.0656–9.223; P = 1.00; potassium: 26.3% *vs* 26.6%, OR 0.982; 95% CI 0.667–3.615; P = 1.00) (Table 3).

**Table 3:** The incidence of symptoms indicating hepatic, renal and bladder toxicity in neuroblastoma patients with poor/intermediate and normal/rapid CYP2B6 metabolizer phenotypes.

	Poor/Intermediate CYP2B6 metabolizers^a^	Normal/Rapid CYP2B6 metabolizers^a^	P value
Hepatotoxicity			
ALT	26.3% (5/19)	60.0% (18/30)	**0.03**
GGT	21.0% (4/19)	40.0% (12/30)	0.21
Renal toxicity			
Creatinine	0.0% (0/19)	6.6% (2/30)	0.51
Sodium	5.2% (1/19)	6.6% (2/30)	1.00
Potassium	26.3% (5/19)	26.6% (8/30)	1.00
Bloody urine	0.0% (0/19)	10% (3/30)	0.27

Significant values are in bold.

^a^Patients’ CYP2B6 phenotypes were predicted on the basis of their *CYP2B6* genotypes.

**Table 4:** Multivariate logistic regression analysis on the incidence of abnormal serum ALT level considering *CYP2B6* SNVs, haplotypes, estimated phenotypes and non-genetic factors (age, sex).

Model	Variables	Increased serum ALT levels		
		Coefficient B (SE)	Exp ß	P
SNVs, non-genetic factors^a^	Constant	− 24.39 (40,192)	0	1
	g.-82T>C (rs34223104)	22.33 (40,192)	4,996,498,070	1
	g.15631G>T (rs3745274)	0.27 (1.37)	1.31	0.84
Model 1	g.18053A>G (rs2279343)	1 (1.39)	0.14	0.16
	g.25505C>T (rs3211371)	− 1.39 (0.89)	0.24	0.11
	Sex	− 0.47 (0.70)	0.95	0.94
	Age	0.02 (0.10)	1.02	0.80
Haplotypes, non-genetic factors^b,d^	Constant	25.60 (79,461)	1.31E+11	1
	CYP2B6*4 (g.-82T/15631G/**18053G**/25505C)	− 0.69 (1.09)	0.49	0.76
	CYP2B6*5 (g.-82T/15631G/18053A/**25505T**)	− 1.22 (0.86)	0.29	0.16
Model 2 (corrected P ≤ 0.01)	CYP2B6*6 (g.-82T/**15631T**/**18053G**/25505T)	− 1.97 (0.74)	0.13	**0.008**
	CYP2B6*22 (g.-**82C**/15631G/18053A/25505C)	− 23.31 (79,461)	7.49E−11	1.00
	Sex	0.21 (0.70)	1.23	0.76
	Age	0.64 (0.71)	1.89	0.37
Estimated phenotype, non-genetic factors^c^	Constant	0.60 (0.75)	1.83	0.42
	Poor/intermediate	− 1.56 (0.67)	0.21	**0.02**
Model 3 (corrected P ≤ 0.02)	Sex	0.11 (0.65)	1.12	0.86
	Age	0.60 (0.68)	1.82	0.38

Significant values are in bold.

^a^AIC: 37.71, BIC: 50.66 for model 1, ^b^AIC: 40.89, BIC: 53.85 for model 2, ^c^AIC: 25.59, BIC: 32.99 for model 3; ^d^Post-hoc power: 94.1%.

In conclusion, mild hepatotoxicity was observed in patients with neuroblastoma receiving cyclophosphamide therapy, which was more frequent in patients with normal/rapid CYP2B6 metabolizing capacity than in those with poor/intermediate CYP2B6 phenotypes. CYP2B6*6 (g.-82T/15631T/18053G/25505T) haplotype seemed to significantly contribute to the decrease of the incidence of abnormal ALT levels during cyclophosphamide therapy. Remarkable kidney and bladder injury was not induced, and no association between CYP2B6 function and the development of renal and bladder toxicity was found in the patients.

### Patients’ *CYP2B6* genotype and the chemotherapy-induced myelosuppression

The most common side effect of cyclophosphamide, as of many other chemotherapeutic agents, is bone marrow suppression; therefore, the association of *CYP2B6* polymorphisms with hematologic toxicity was also assessed. All patients suffered from hematologic toxicity induced by the therapy, and the decrease in blood cell counts generally developed by the 7th–15th day after treatment, in line with the observations of previous studies^10,17,70,71^. Grade 3 lymphopenia, grade 4 neutropenia and grade 3 or 4 thrombocytopenia developed most frequently, and in the majority of the patients, the cell counts of monocytes, eosinophil granulocytes and red blood cells were lower than those in the normal reference populations (Supplementary Table 2, Table 5). However, the severity and incidence rates varied in patients with poor/intermediate and normal/rapid CYP2B6 metabolizing capacities (Table 5, Supplementary Fig. 1). Grade 3 lymphopenia occurred more frequently in patients with normal/rapid function than in those with poor/intermediate CYP2B6 function (Chi^2^: 6.044, N = 47, P = 0.015). The prevalence of severe thrombocytopenia (grades 3 and 4) was also higher in normal/rapid CYP2B6 metabolizers than in poor/intermediate metabolizer patients (Chi^2^: 5.588, N = 50, P = 0.018). Furthermore, chemotherapy-induced decrease in monocyte counts developed significantly more frequently in patients with normal/rapid than in those with poor/intermediate CYP2B6 function (OR 10.000; 95% CI 1.056–94.730; N = 45, P = 0.030). However, no association between CYP2B6 metabolizing capacity and the decrease in cell counts of neutrophil and eosinophil granulocytes as well as of red blood cells was observed in the patients during the 3-cycle therapy containing cyclophosphamide. In conclusion, the patients’ CYP2B6 metabolizing capacity appeared to significantly influence the development of severe hematologic toxicity related to lymphopenia, thrombocytopenia and monocytopenia induced by the therapy, whereas no effect of CYP2B6 function on the reduction of neutrophils, eosinophils and red blood cells was observed in neuroblastoma patients.

**Table 5:** Evaluation of hematologic parameters indicating myelosuppression in patients with poor/intermediate and normal/rapid CYP2B6 function.

	Poor/intermediate CYP2B6 metabolizers	Normal/rapid CYP2B6 metabolizers	P value
Lymphocytes (N = 47)			**0.015**
Grade 0	5.6% (1/18)	0.0% (0/29)	
Grade 1/2	22.2% (4/18)	3.4% (1/29)	
Grade 3	72.2% (13/18)	96.6% (28/29)	
Platelets (N = 50)			**0.018**
Grade 0	35.0% (7/20)	10.0% (3/30)	
Grade 1/2	15.0% (3/20)	10% (3/30)	
Grade 3/4	50.0% (10/20)	80.0% (24/30)	
Neutrophils (N = 49)			0.306
Grade 0	0.0% (0/19)	0.0% (0/30)	
Grade 1/2	10.5% (2/19)	3.3% (1/30)	
Grade 3/4	89.5% (17/19)	96.7% (29/30)	
Monocytes (N = 45)			**0.030**
> LLN*	27.8% (5/18)	3.7% (1/27)	
< LLN*	72.2% (13/18)	96.3% (26/27)	
Eosinophils (N = 45)			0.449
> LLN*	27.8% (5/18)	14.8% (4/27)	
< LLN*	72.2% (13/18)	85.2% (23/27)	
Red blood cells (N = 50)			0.489
> LLN*	15.0% (3/20)	26.7% (8/30)	
< LLN*	85.0% (17/20)	73.3% (22/30)	

Significant values are in bold.

*LLN: lower limit in normal reference population at the same age.

### Association between patients’ *CYP* genotypes and therapeutic outcome

**Figure 1:**
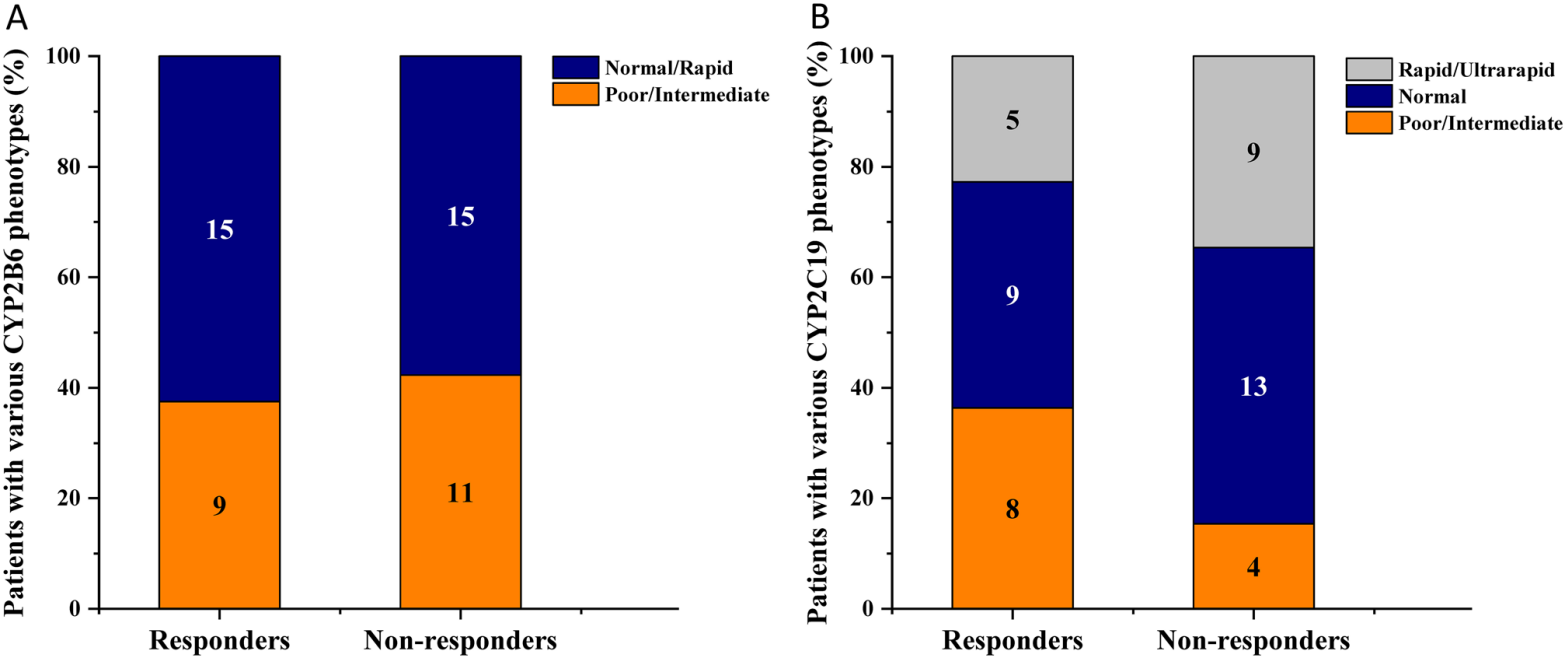
The association of cyclophosphamide therapeutic outcome with patients’ CYP2B6 (**A**) and CYP2C19 genotype-based phenotypes (**B**). Patients were considered to be responders with complete remission and partial remission, whereas non-responders with stable disease and progressive disease/exit. CYP2B6 and CYP2C19 phenotypes were estimated on the basis of *CYP2B6* or *CYP2C19* genotypes according to the CPIC and PharmVar recommendations.

Although CYP2B6 plays a major role and CYP2C19 is the minor catalyst of cyclophosphamide metabolism, the association of the drug-metabolizing capacity of both CYP enzymes with therapeutic outcomes was retrospectively evaluated. CYP2B6 and CYP2C19 phenotypes of the patients were established on the basis of their genotypes, whereas the patients’ response to anticancer therapy was defined as responders (complete remission and partial remission, N = 24/50) and non-responders (stable disease and progressive disease/exit, N = 26/50) on the basis of the primary tumour response (Tables 1 and 2). No significant differences were observed in the ratios of patients with various CYP2B6 or CYP2C19 phenotypes between responders and non-responders (CYP2B6 poor/intermediate : normal/rapid metabolizers 9:15 in responders and 11:15 in non-responders, P > 0.05; CYP2C19 poor/intermediate : normal : rapid/ultrarapid metabolizers 8:9:5 in responders and 4:13:9 in non-responders, P > 0.05) (Fig. 1). Multivariate binary logistic regression analysis was also performed to estimate the influence of *CYP2B6* SNVs and haplotypes as well as of non-genetic factors, including sex and age, on treatment outcomes (Table 6). Although *CYP2B6* genetic variability appeared to display no association with patients’ response to chemotherapy, a significant contribution of sex and age to therapeutic outcomes was demonstrated. According to the model with *CYP2B6* SNVs or haplotypes, the primary tumour response was better in patients under 1.5 years than in older children, and girls were found to expect more favourable therapeutic outcomes than boys in both models (P = 0.03) (Table 6).

**Table 6:** Multivariate logistic regression analysis on response to cyclophosphamide therapy considering *CYP2B6* genetic polymorphisms, estimated phenotypes and non-genetic factors (age, sex).

Models	Variables	Response to therapy		
		Coefficient B (SE)	Exp ß	P value
SNVs, non-genetic factors^a,e^	Constant	23.31 (40,192)	1.338E+10	1.00
	g.-82T>C (rs34223104)	− 22.11 (40,192)	0.00	1.00
	g.15631G>T (rs3745274)	0.29 (1.47)	1.34	0.83
Model 1 (corrected P ≤ 0.01)	g.18053A>G (rs2279343)	0.45 (1.45)	1.58	0.75
	g.25505C>T (rs3211371)	0.38 (0.9)	1.46	0.67
	Sex	− 1.5 (0.72)	0.22	0.03
	Age	− 0.75 (0.74)	0.17	**0.01**
Haplotypes^d^, non-genetic factors^b,e^	Constant	21.83 (40,192)	3,041,354,571	1.00
	CYP2B6*4 (g.-82T/15631G/**18053G**/25505C)	1.39 (1.22)	4.01	0.25
	CYP2B6*5 (g.-82T/15631G/18053A/**25505T**)	0.52 (0.91)	1.68	0.56
Model 2 (corrected P ≤ 0.02)	CYP2B6*6 (g.-82T/**15631T**/**18053G**/25505T)	0.73 (0.73)	2.08	0.32
	CYP2B6*22 (g.-**82C**/15631G/18053A/25505C)	− 21.99 (40,192)	0.00	1.00
	Sex	− 1.57 (0.73)	0.20	0.03
	Age	− 1.7 (0.75)	0.18	**0.02**
Estimated phenotype, non-genetic factors^c,e^	Constant	− 1.39 (0.77)	0.24	0.07
	Poor/intermediate	− 0.69 (0.68)	0.49	0.3
Model 3 (corrected P ≤ 0.02)	Sex	1.36 (0.69)	3.89	0.04
	Age	1.58 (0.71)	4.86	**0.02**

Significant values are in bold.

^a^AIC: 41.63, BIC: 54.72 for model 1, ^b^AIC: 44.11, BIC: 57.21 for model 2, ^c^AIC: 25.46, BIC: 32.94, ^d^In haplotypes, the polymorphic variants were indicated in bold. ^e^Post-hoc power: 57.0%.

## Discussion

CYP2B6 is considered to be the major catalyst of CYP-mediated activation of cyclophosphamide, and a minor role is attributed to CYP2C19, whereas the inactivation pathways by these enzymes also lead to the formation of toxic metabolites, such as acrolein and chloroacetaldehyde^10^. Consequently, the remarkable genetic variability of *CYP2B6* and *CYP2C19* is likely to influence the patients’ response to cyclophosphamide, resulting in differences in therapeutic efficacy and development of side effects^30^. Therefore, pharmacogenetic testing may facilitate justification of cyclophosphamide-induced adverse events or weak therapeutic efficacy. However, a clear pharmacogenetic evidence for chemotherapeutic drugs in pediatric patients is limited, and the interpretation of pharmacogenetic data in children may be assisted by extrapolation from adults^72,73^. Although developmental expression patterns of several drug-metabolizing enzymes leading to different drug responses in children and adults have been reported^74^, the consequences of *CYP2B6* genetic variants in pediatric patients are expected to be identical to those in adults, because CYP2B6 expression rapidly increases after birth and is constant after 1 year of age^51,73^. In contrast, the activity and protein expression of CYP2C19 is low in young children and approaches the adult level only after 10 to 18 years of age^75^. Thus, in pediatric patients, CYP2B6 catalyzed oxidation may become the principal route of cyclophosphamide metabolism, and *CYP2B6* genetic polymorphisms may influence the predisposition to cyclophosphamide-induced adverse reactions.

The active metabolite phosphoramide mustard and toxic byproducts of cyclophosphamide metabolism induce clinically significant organ-specific side effects^10,17^. The present study focused on the impact of *CYP2B6* genetic variability on the development of liver and excretory system (kidneys and urinary bladder) injury as well as of hematologic toxicity in pediatric patients undergoing cyclophosphamide therapy. In these neuroblastoma patients, a significant association of *CYP2B6* genetic variants with liver injury and hematologic toxicity was observed, but not with renal and bladder injury. Poor/intermediate CYP2B6 metabolizers carrying at least one CYP2B6*6 allele and having reduced CYP2B6 activity were assumed to produce low levels of toxic phosphoramide mustard and acrolein due to diminished cyclophosphamide metabolism, that might explain the low incidence of liver and hematologic toxicity. Although, neuroblastoma considered to be the most prevalent extracranial solid tumour malignancy in childhood^1,2^, cyclophosphamide-induced toxicity related to the patients’ CYP2B6 and CYP2C19 status has not been extensively investigated in patients with neuroblastoma. In the literature, cyclophosphamide-induced toxicity has been evaluated in patients with tumour malignancies other than neuroblastoma; therefore, our findings interpreting *CYP* polymorphisms and cyclophosphamide-related adverse reactions in pediatric patients with neuroblastoma appear to be the first. The rate of cyclophosphamide metabolism is considered to be an important factor in cyclophosphamide-induced toxicity^10^, and our results highlighted that estimating patients’ cyclophosphamide metabolizing capacity by identification of clinically relevant *CYP* polymorphisms may predict cyclophosphamide-induced adverse reactions even in neuroblastoma patients. Our results were in line with the findings in Japanese breast cancer patients receiving a standard AC regimen (doxorubicin and cyclophosphamide) that grade 4 neutropenia hardly developed in CYP2B6*6 carriers^55^. However, other studies found no association of hematologic toxicity with pharmacokinetics of cyclophosphamide and its metabolites or with *CYP2B6* polymorphisms^19,34,53,57^. Increased hydroxylation activity forming 4-hydroxycyclophosphamide was linked to a significant reduction of neutrophils and platelets and to low hemoglobin concentrations in pediatric patients with brain tumours that also confirmed the association between high bioactivation rate and increased risk of hematologic toxicity^34^. Transient elevation of serum bilirubin, alkaline phosphatase, ALT or aspartate aminotransferase levels has also been reported in patients undergoing chemotherapy with cyclophosphamide^76,77^; however, no link to *CYP2B6* genotype has been established^52^.

Sensitivity to cyclophosphamide appears to be increased in cells with reduced detoxification by aldehyde dehydrogenase (ALDH enzyme). ALDH1A1 and ALDH3A1 enzymes catalyze the conversion of aldophosphamide, the precursor of phosphoramide mustard, to carboxyphosphamide, which has no alkylating and cytotoxic activity^17^. Liver cells are less sensitive to cyclophosphamide because of the high level of ALDH^16^, that might explain the mild hepatotoxicity observed in patients with neuroblastoma in the present study (Supplementary Table 2). However, Ming et al. reported severe and prolonged hepatotoxicity in a breast cancer patient after two cycles of cyclophosphamide, which was attributed to the combined effect of the *CYP2B6* variant with high cyclophosphamide 4-hydroxylation activity and the *ALDH3A1* genetic variant with reduced detoxification activity^78^. Hematopoietic progenitors or lymphocyte subsets express low levels of ALDH, predisposing these cells to have a low inactivation rate and to be more sensitive to the bioactivated compound; therefore, enhanced hematologic toxicity is expected to emerge^16,79,80^ as it was found in neuroblastoma patients (Supplementary Table 2). Severe (grade 3 and grade 4) hematologic toxicity (leukocytopenia, and neutropenia) was observed in more than half of the adult Japanese cancer patients treated with cyclophosphamide, and grade 4 toxicity was associated with high 4-hydroxycyclophosphamide exposure and *CYP2B6* polymorphisms^33^. Hematologic toxicity of cyclophosphamide has also been reported in 12–48% of pediatric patients with solid tumour malignancies^35^, whereas hemorrhagic cystitis or excretory system-related toxicities are relatively uncommon after cyclophosphamide administration^52,76,77,81^. Although Muniz et al. reported an association between CYP2B6*4 and hemorrhagic cystitis in adult patients treated with high-dose cyclophosphamide, according to our findings in patients with neuroblastoma, bloody urine symptoms were rarely observed^82^.

Conflicting results have been reported regarding patients’ responses to cyclophosphamide-containing chemotherapy. Several studies have revealed a significant association of favourable or unfavourable treatment outcomes with *CYP2B6* or *CYP2C19* pharmacogenetics, whereas others have hardly demonstrated any relationship between treatment response and *CYP* polymorphisms^44,53,54,83–86^. According to Pinto et al., the 3-year event-free survival of rhabdomyosarcoma patients after vincristine/actinomycin/cyclophosphamide (VAC) therapy was not related to any SNVs in drug-metabolizing enzymes, including *CYP2B6* and *CYP2C19*, whereas Labib et al. revealed favourable therapeutic outcomes in patients carrying the CYP2B6 K262R variant with the same malignancy and therapy protocol^54,86^. The cyclophosphamide therapy in the neuroblastoma patients of the present study followed the treatment protocols considering patients’ age, bodyweight and risk stratification. It should be noted that their chemotherapy regimen contained additional anticancer drugs, such as vincristine, adriamycin, cisplatin, carboplatin or etoposide, according to pretreatment risk stratification and risk-adapted therapeutic protocols. The overall response rate was found to be associated with age categorized as younger and older than 1.5 years, and a trend was also observed toward significance with sex; however, neither the *CYP2B6* or *CYP2C19* haplotypes nor genotype-based phenotypes had an influence on therapeutic outcomes in neuroblastoma patients. Patients’ age at the time of neuroblastoma diagnosis is one of the most important risk factors for risk stratification. Children under 1–1.5 years of age can expect better treatment outcomes than older subjects, regardless of their favourable or unfavourable disease staging^5,87–90^. Gender is not considered to be a prognostic factor in risk stratification^87,88,91^; however, several studies have indicated that female patients can expect more favourable prognosis according to survival analysis^89,90,92^. The SIOP Europe Neuroblastoma Group study demonstrated that boys with stage 1 disease without MYCN (v-myc myelocytomatosis viral related oncogene, neuroblastoma derived) gene amplification suffered more relapse episodes during the 5-year follow-up period than did female patients^93^.

The present study had some limitations. First, both low- and high-risk neuroblastoma patients were included in this study; therefore, various cyclophosphamide-containing chemotherapy regimens were applied. However, the distribution of low- and high-risk subjects in the CYP2B6 and CYP2C19 metabolizer groups was homogenous. Second, the association of the development of adverse reactions and therapeutic responses with the genetic variability of CYP enzymes responsible for the first steps of cyclophosphamide metabolism was established, and other enzymes catalyzing the subsequent metabolic steps were not evaluated. Third, the toxicity data of the patients were retrospectively analysed, and adverse reaction data could not be systematically collected.

## Conclusion

Although the pharmacokinetic variability of cyclophosphamide and its toxic byproducts is well documented in adults^20,30–34^, the potential contribution of genetic polymorphisms of CYP enzymes involved in cyclophosphamide metabolic pathways to the treatment outcome and development of side effects is not completely clear. In the present study, the contribution of pharmacogenetic variability in *CYP2B6* and *CYP2C19* to treatment efficacy and cyclophosphamide-induced side effects was evaluated in pediatric patients with neuroblastoma. Cyclophosphamide-induced hepatorenal toxicity was mild, whereas hematologic toxicities were severe and occurred in all patients. After multiple cycles of cyclophosphamide treatments, the incidence of liver injury and hematologic toxicities, including lymphopenia, thrombocytopenia and monocytopenia, but not excretory system (kidneys, urinary bladder) toxicities were associated with the patients’ CYP2B6 metabolizer phenotype. Furthermore, the therapeutic response to cyclophosphamide appeared to depend on the patients’ age and gender; however, CYP2B6 or CYP2C19 metabolizer phenotypes did not influence the treatment outcome. Our results may contribute to a better understanding of the impact of CYP2B6 variability on cyclophosphamide-induced side effects.

## Supplementary Information

Supplementary Information.

## Data Availability

The data that support the findings of this study have been deposited in the European Variation Archive (EVA)^94^ at EMBL-EBI under accession number PRJEB61781 (https://www.ebi.ac.uk/eva/?eva-study=PRJEB61781).

## Abbreviations

ALDH: Aldehyde dehydrogenase
ALT: Alanine aminotransferase
CPIC: Clinical Pharmacogenetics Implementation Consortium
GGT: Gamma-glutamyltransferase
CTC: Common toxicity criteria
CYP: Cytochrome P450
INRC: International Neuroblastoma Response Criteria
INSS: International Neuroblastoma Staging System
PharmVar: Pharmacogene Variation Consortium
MYCN: V-Myc myelocytomatosis viral related oncogene, neuroblastoma derived
SNV: Single nucleotide variation
