# Supplementary material for: Association between CYP2B6 genetic variability and cyclophosphamide therapy in pediatric patients with neuroblastoma
Source: Sci Rep. 2023 Jul 21;13:11770. doi: 10.1038/s41598-023-38983-0 (PMC10361978; doi:10.1038/s41598-023-38983-0)
Supplement: Supplementary file 1 — Supplementary Information. [file 41598_2023_38983_MOESM1_ESM.docx]

**Supplementary table 1.** Clinical data, *CYP2B6* and *CYP2C19* genotypes of neuroblastoma patients

| **Patient ID** | **Age (year)** | **Gender** | **MYCN Status** | **Treatment Outcome*** | **CYP2B6 genotype** | **CYP2C19 genotype** |
| --- | --- | --- | --- | --- | --- | --- |
| SAMPLE_001 | 1.16 | Male | Positive | PD-Exit | *1/*4 | *1/*1 |
| SAMPLE_002 | 7.93 | Male | Negative | PD-Exit | *6/*6 | *1/*17 |
| SAMPLE_003 | 2.27 | Female | Negative | CR | *1/*6 | *2/*17 |
| SAMPLE_004 | 1.34 | Male | Negative | CR | *1/*1 | *1/*1 |
| SAMPLE_005 | 2.40 | Male | Positive | CR | *1/*22 | *2/*2 |
| SAMPLE_006 | 3.63 | Female | Negative | PD-Exit | *1/*1 | *1/*1 |
| SAMPLE_007 | 4.60 | Female | Negative | CR | *1/*6 | *1/*1 |
| SAMPLE_008 | 8.65 | Male | Negative | PD-Exit | *1/*1 | *17/*17 |
| SAMPLE_009 | 0.28 | Male | Negative | SD | *1/*6 | *1/*1 |
| SAMPLE_010 | 8.70 | Female | Negative | SD | *1/*5 | *1/*1 |
| SAMPLE_011 | 1.45 | Male | Negative | SD | *1/*6 | *1/*17 |
| SAMPLE_012 | 3.70 | Male | Negative | SD | *1/*1 | *1/*2 |
| SAMPLE_013 | 1.27 | Male | Negative | PR | *1/*1 | *1/*1 |
| SAMPLE_014 | 2.53 | Male | Negative | SD | *1/*1 | *1/*1 |
| SAMPLE_015 | 0.96 | Female | Positive | PD-Exit | *5/*6 | *1/*17 |
| SAMPLE_016 | 3.16 | Male | Negative | PD-Exit | *1/*1 | *1/*17 |
| SAMPLE_017 | 2.12 | Female | Positive | CR | *1/*6 | *1/*1 |
| SAMPLE_018 | 0.20 | Female | Negative | PR | *1/*5 | *1/*1 |
| SAMPLE_019 | 1.86 | Male | Positive | SD | *1/*4 | *1/*1 |
| SAMPLE_020 | 4.53 | Female | Negative | PR | *1/*5 | *2/*2 |
| SAMPLE_021 | 1.77 | Female | Positive | SD | *4/*6 | *2/*17 |
| SAMPLE_022 | 4.45 | Male | Negative | PR | *1/*4 | *1/*1 |
| SAMPLE_023 | 0.87 | Female | Positive | CR | *1/*5 | *1/*1 |
| SAMPLE_024 | 2.53 | Male | Negative | CR | *1/*5 | *1/*1 |
| SAMPLE_025 | 0.10 | Male | Positive | CR | *1/*1 | *1/*2 |
| SAMPLE_026 | 5.30 | Male | Negative | SD | *1/*1 | *1/*17 |
| SAMPLE_027 | 2.41 | Male | Positive | PD-Exit | *1/*1 | *1/*1 |
| SAMPLE_028 | 2.55 | Female | NA* | PD-Exit | *1/*1 | *1/*2 |
| SAMPLE_029 | 3.42 | Male | Positive | PD-Exit | *5/*6 | *1/*17 |
| SAMPLE_030 | 0.75 | Male | Positive | PD-Exit | *1/*6 | *1/*17 |
| SAMPLE_031 | 0.90 | Male | Negative | CR | *1/*5 | *1/*1 |
| SAMPLE_032 | 1.89 | Female | Positive | PD-Exit | *4/*6 | *1/*1 |
| SAMPLE_033 | 2.36 | Male | Negative | PD-Exit | *1/*6 | *1/*17 |
| SAMPLE_034 | 15.81 | Female | Negative | SD | *1/*5 | *1/*1 |
| SAMPLE_035 | 2.94 | Female | Positive | PD-Exit | *1/*6 | *1/*1 |
| SAMPLE_036 | 1.24 | Female | Negative | PR | *6/*6 | *2/*17 |
| SAMPLE_037 | 4.89 | Female | Negative | CR | *1/*1 | NA* |
| SAMPLE_038 | 1.61 | Male | Positive | PD-Exit | *1/*5 | *1/*1 |
| SAMPLE_039 | 3.06 | Female | Positive | CR | *1/*6 | *1/*17 |
| SAMPLE_040 | 1.91 | Male | Positive | PD-Exit | *1/*1 | *1/*1 |
| SAMPLE_041 | 12.28 | Female | Negative | CR | *1/*1 | *1/*2 |
| SAMPLE_042 | 0.24 | Female | Positive | CR | *1/*1 | *1/*17 |
| SAMPLE_043 | 5.24 | Male | Negative | PD-Exit | *1/*6 | *1/*1 |
| SAMPLE_044 | 6.41 | Male | Negative | CR | *1/*1 | *17/*17 |
| SAMPLE_045 | 6.02 | Male | Negative | PR | *1/*6 | *1/*2 |
| SAMPLE_046 | 0.18 | Male | Negative | CR | *1/*6 | *1/*17 |
| SAMPLE_047 | 0.45 | Male | Negative | PR | *1/*6 | *1/*2 |
| SAMPLE_048 | 2.01 | Male | Positive | PD-Exit | *1/*5 | *2/*2 |
| SAMPLE_049 | 0.84 | Female | Negative | CR | *1/*6 | *1/*1 |
| SAMPLE_050 | 1.88 | Female | Negative | PR | *1/*1 | *17/*17 |

*NA: data not available, CR complete remission, PR partial remission, SD stable disease, PD/Exit progressive disease/exit

**Supplementary table 2.** Grades of hepatic, renal and blood/bone marrow toxicities in neuroblastoma patients.

| **Parameters^a^** | **Grade 1** | **Grade 2** | **Grade 3** | **Grade 4** |
| --- | --- | --- | --- | --- |
| **Hepatic function** |  |  |  |  |
| ALT^b^ | 18/49 | 4/49 | 1/49 | - |
| GGT^b^ | 12/49 | 3/49 | 1/49 | **-** |
| **Renal function** |  |  |  |  |
| Creatinine^c^ | 2/49 | - | - | - |
| **Hematologic parameters** |  |  |  |  |
| Lymphocytes^d^ | 3/47 | 2/47 | 41/47 | - |
| Neutrophil granulocytes^e^ | 2/49 | 1/49 | 4/49 | 42/49 |
| Platelets^f^ | 3/50 | 3/50 | 23/50 | 11/50 |

^a^based on National Cancer Institute Common Toxicity Criteria (CTC) version 2.0; ULN: upper limit in normal reference population; LLN: lower limit limit in normal reference population

^b^Grade 1: >ULN-2.5xULN; Grade 2: >2.5-5.0xULN; Grade 3: >5.0-20.0xULN; Grade 4: >20.0xULN

^c^Grade 1: >ULN-1.5xULN; Grade 2: >1.5-3.0xULN; Grade 3: >3.0-6.0xULN; Grade 4: >6.0xULN

^d^Grade 1: <LLN-1.0x10^9^/L; Grade 2: >0.5-1.0x10^9^/L; Grade 3: <0.5x10^9^/L

^e^Grade 1: >1.5-2.0x10^9^/L; Grade 2: >1.0-1.5x10^9^/L; Grade 3: >0.5-1.0x10^9^/L; Grade 4: <0.5x10^9^/L

^f^Grade 1: <LLN-75x10^9^/L; Grade 2: ≥50-<75x10^9^/L; Grade 3: ≥10-<50x10^9^/L; Grade 4: <10x10^9^/L


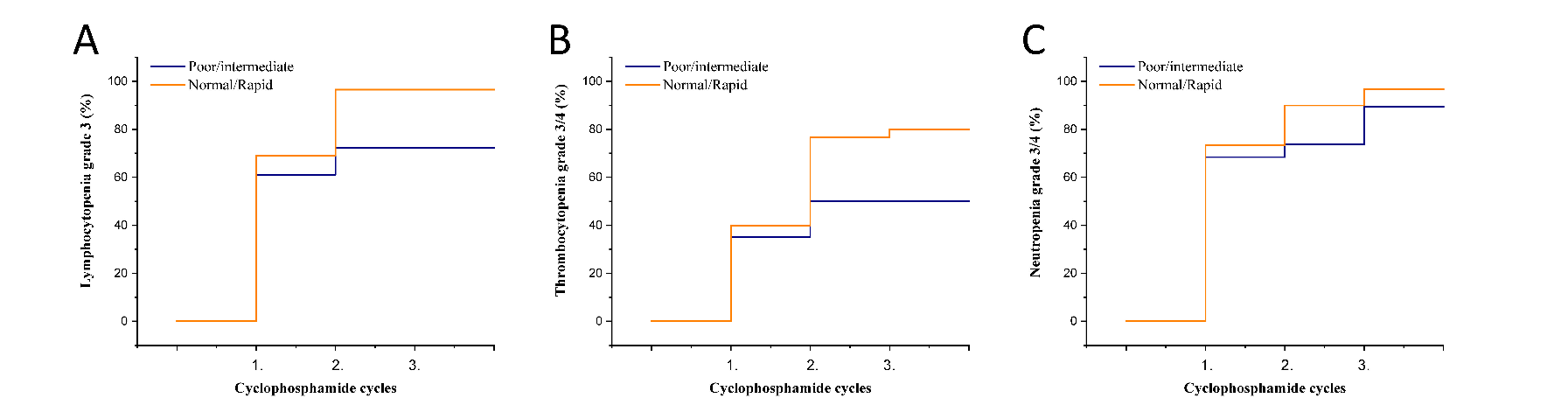


**Supplementary figure 1.** The incidence of lymphopenia (A), thrombocytopenia (B) and neutropenia (C) in neuroblastoma patients with poor-intermediate and normal-rapid CYP2B6 metabolizing capacity during three cycles of cyclophosphamide containing therapy.
